# Health-enhancing physical activity during leisure time among adults in Germany

**DOI:** 10.17886/RKI-GBE-2017-040

**Published:** 2017-06-14

**Authors:** Jonas D. Finger, Gert B. M. Mensink, Cornelia Lange, Kristin Manz

**Affiliations:** Robert Koch Institute, Department for Epidemiology and Health Monitoring, Berlin, Germany

**Keywords:** PHYSICAL ACTIVITY, PHYSICAL ACTIVITY RECOMMENDATIONS, ADULTS, GERMANY, HEALTH MONITORING

## Abstract

Self-reported data from the GEDA 2014/2015-EHIS study was used to calculate the level of compliance among adults in Germany with the World Health Organization’s (WHO) recommendations on physical activity. The WHO’s recommendations distinguish between ‘aerobic activity’ and ‘muscle-strengthening activity’. In Germany, 42.6% of women and 48.0% of men reported that they conduct at least 2.5 hours of aerobic physical activity per week, and therefore meet the WHO’s recommendation on this form of activity. A higher level of education among women and men of all ages is associated with a higher frequency of meeting the WHO’s recommendations on aerobic activity. In addition, 27.6% of women and 31.2% of men conduct muscle-strengthening activity at least twice a week, thereby meeting the WHO’s recommendations on this form of activity. About one fifth of women (20.5%) and one quarter of men in Germany (24.7%) meet both of these recommendations. In summary, the results point to the value of encouraging people to conduct more physical activity during their leisure time. In fact, inactive people who begin to follow the WHO’s recommendations can significantly reduce their long-term risk of premature mortality.

## Introduction

Physical activity is defined as any bodily movement generated by the skeletal muscles that requires more energy to be consumed than the basal metabolic rate [1]. Health-enhancing physical activity includes aerobic activity (endurance activity) such as cycling, jogging, playing football or swimming that increases breathing and heart rate and is undertaken without interruption for at least 10 minutes [2]. Aerobic activity provides an important contribution to the maintenance and recovery of the health of the cardiovascular and metabolic system [3, 4]. Muscle-strengthening activity such as strength training, Pilates and yoga is also beneficial to health, as it increases the performance and health of the human musculoskeletal system, skeletal muscles, joints, bones, tendons and ligaments [2, 4]. In contrast, a lack of physical activity increases the risks of developing the most important non-communicable diseases such as heart disease, type 2 diabetes mellitus, and breast and colorectal cancer, and it also reduces life expectancy [5]. According to the 2015 Global Burden of Disease Study, physical inactivity in Germany contributes to a significant reduction in life expectancy and quality of life. Specifically, physical inactivity was found to be linked to 10% of the years lost due to coronary heart disease, 17% of the years lost due to diabetes mellitus, 15% of the years lost due to colorectal cancer and 10% of the years lost due to breast cancer [6]. As insufficient levels of physical activity are associated with disease, the World Health Organization (WHO) formulated the goal of reducing the prevalence of insufficient physical activity (defined as less than 2.5 hours of moderate- to vigorous-intensity physical activity per week) as part of the Global Action Plan for the Prevention and Control of Non-Communicable Diseases 2013-2020. The aim is to ensure that levels of insufficient physical activity are 10% lower than 2010 levels by 2025 [7].

**GEDA 2014/2015-EHIS**

**Data holder:** Robert Koch Institute

**Aims:** To provide reliable informa tion about the population’s health status, health-related behaviour and health care in Germany, with the possibility of a European comparison

**Method:** Questionnaires completed on paper or online

**Population:** People aged 18 years and above with permanent residency in Germany

**Sampling:** Registry office sample; randomly selected individuals from 301 communities in Germany were invited to participate

**Participants:** 24,016 people (13,144 women; 10,872 men)

**Response rate:** 26.9%

**Study period:** November 2014 - July 2015

**Data protection:** This study was undertaken in strict accordance with the data protection regulations set out in the German Federal Data Protection Act and was approved by the German Federal Commissioner for Data Protection and Freedom of Information. Participation in the study was voluntary. The participants were fully informed about the study’s aims and content, and about data protection. All participants provided written informed consent.

More information in German is available at www.geda-studie.de

## Indicator

The WHO’s recommendations on physical activity differentiate between ‘aerobic activity’ and ‘muscle-strengthening activity’ [2, 8]. Adherence to these recommendations among the population in Germany [8] was assessed with the validated German version of the European Health Interview Survey – Physical Activity Questionnaire (EHIS-PAQ) used for the German Health Update (GEDA 2014/2015-EHIS) survey [9, 10]. As part of this study, respondents were asked about the duration of the physical activity they undertake during a typical week, in the form of both moderate-intensity aerobic physical activity conducted during leisure time and cycling used for transportation, as well as the number of days a week during which they undertake muscle-strengthening activities. Details about the way in which these indicators were constructed have been published elsewhere [10]. The following describes the proportion of respondents who conduct at least moderate-intensity aerobic activities for at least 2.5 hours a week (the first part of the WHO’s recommendations on physical activity), as well as those who conduct muscle-strengthening activities on at least two days a week (the second part of the WHO’s recommendations), and the proportion of those who meet both parts of the WHO’s recommendations (2.5 hours of aerobic activity, as well as muscle-strengthening activities twice a week). The figures are stratified according to gender, age, level of education and federal state. A difference between these groups is interpreted as statistically significant where confidence intervals do not overlap.

The analyses are based on data from 22,959 participants aged 18 years and above (12,511 women, and 10,448 men) with valid EHIS-PAQ data. The calculations were carried out using a weighting factor that corrects for deviations within the sample from the structure of the German population (as of 31 December 2014) with regard to gender, age, community type and education. The community type reflects the degree of urbanisation and corresponds to the regional distribution in Germany. The International Standard Classification for Education (ISCED) was used to ensure the respondents’ responses on education were comparable [11]. A detailed description of the methodology applied in GEDA 2014/2015-EHIS can be found in the article German Health Update: New data for Germany and Europe in issue 1/2017 of the Journal of Health Monitoring.

## Results and discussion

According to results from the GEDA 2014/2015-EHIS study, 42.6% of women and 48.0% of men meet the WHO’s recommendation on aerobic activity (Tables 1 and Table 2). 56.7% of men aged between 18 and 29 meet the WHO’s recommendations on aerobic activity; the same can be said of around 45% of men in other age groups. Among women, compliance with the recommendations is highest in the 45-to-64 age group (47.8%). No uniform pattern can be observed among women in terms of age distribution. A smaller proportion of women (27.6%) and men (31.2%) meet the WHO’s recommendation on muscle-strengthening activity. About one fifth of women (20.5%) and one quarter of men (24.7%) meet both recommendations.

An association exists between level of education and health-enhancing aerobic physical activity among women and men of all age groups: the proportion of adults who meet the recommendations on physical activity is lower in groups with lower levels of education compared to those with the higher levels of education (Table 1 and Table 2).

**Figure 1:**
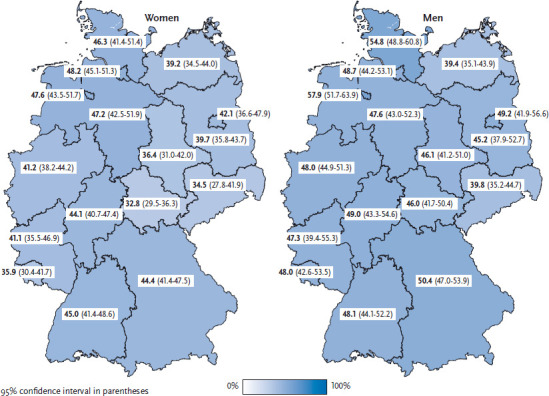
Proportion of women and men complying with the World Health Organization’s recommendation on aerobic activity according to German federal state (n=12,511 women; n=10,448 men) Source: GEDA 2014/2015-EHIS

The proportion of women in Thuringia who meet the recommendations on aerobic activity is below the national average; in Hamburg, it is above the national average. The proportion of men in Mecklenburg-West Pomerania and Saxony who meet the recommendations on aerobic activity is below the national average; in Bremen, it is above the national average (Figure 1).

The research that formed the basis of the WHO’s recommendations on aerobic activity leads to the conclusion that people who undertake moderate- to vigorious-intensity aerobic activities for at least 2.5 hours per week have a significantly lower risk of all-cause mortality [4]. However, there is no absolute threshold in terms of risk reduction: some physical activity is good; more physical activity is better [4]. In fact, the most active group has an estimated 30% lower risk of premature mortality than the least active group [4].

Nevertheless, the calculations on compliance with the WHO’s recommendations on aerobic activity only consider aerobic activity and transport-related cycling that is undertaken during leisure time; it does not include work-related physical activity [10]. This is important because population groups that are less likely to follow the recommendations on aerobic activity, such as adults with lower levels of education, are generally engaged in more physically active forms of employment [12, 13]. This is also confirmed when the regional differences between work-related and leisure-time physical activity are compared by federal state: in states where a high proportion of people undertake leisure-time physical activity (such as Hamburg), a lower proportion conducts high levels of work-related activity. In contrast, states that demonstrate a high level of work-related activity (such as Thuringia) tend to have a lower proportion of people engaging in leisure-time physical activity [13]. Be this as it may, work-related physical activity does not usually provide the same health benefits as aerobic physical exercise conducted during leisure time [14, 15].

It is not possible to use the results from the GEDA 2014/2015-EHIS study and those of previous GEDA waves to calculate time trends because the physical activity questionnaire was changed. The GEDA 2014/2015-EHIS study used EHIS-PAQ, a new survey instrument. EHIS-PAQ was developed in 2010 in order to estimate the compliance with the WHO’s recommendations.

Still, another study conducted in Germany confirms that about half of adults in Germany meet the WHO’s recommendations [16]. However, again the results of this survey and those of the GEDA 2014/2015-EHIS study can only be compared to a limited extent due to the use of different survey instruments.

Overall, the results set out here point to the importance of encouraging people to conduct more physical activity during their leisure time. More than half of the adult population undertakes less than 2.5 hours per week of at least moderate-intensity aerobic physical activity, and thus fails to meet the core aspect of the WHO’s recommendations on physical activity. In view of the costs incurred due to physical inactivity (through time taken off work, illness and premature mortality) [17], increased investment in measures that encourage people to be more physically active is both sensible and necessary. This could include population-based informational approaches, community-based intervention, and political and environmental approaches undertaken within the framework of the German national recommendations on physical activity and physical activity promotion [18].

## Key statements

- 42.6% of women and 48.0% of men in Germany meet the World Health Organization’s recommendations on aerobic activity by undertaking at least 2.5 hours of aerobic physical activity per week.
- 27.6% of women and 31.2% of men in Germany meet the World Health Organization’s recommendations on muscle-strengthening activity by conducting muscle-strengthening activity at least twice a week.
- Women meet the World Health Organization’s recommendations on aerobic activity statistically significantly less often than men.
- A higher level of education among women and men of all ages is associated with a higher frequency of meeting the World Health Organization’s recommendations on aerobic activity.

## Figures and Tables

**Table 1:** Health-enhancing physical activity during leisure time among women according to age and educational status (n=12,511) Source: GEDA 2014/2015-EHIS

Women	Aerobic activity at least 2.5 hours per week		Muscle-strengthening activity at least 2 times a week		Aerobic and muscle-strengthening activity recommendations compliance	
	%	(95% CI)	%	(95% CI)	%	(95% CI)
**Women total**	**42.6**	**(41.3-43.9)**	**27.6**	**(26.7-28.6)**	**20.5**	**(19.6-21.4)**
**18-29 Years**	45.2	(42.3-48.2)	34.5	(32.1-37.0)	25.8	(23.6-28.2)
Low education	40.1	(34.2-46.3)	29.5	(24.3-35.3)	21.9	(17.2-27.3)
Medium education	44.4	(40.6-48.2)	35.8	(32.6-39.1)	26.0	(23.1-29.1)
High education	55.0	(49.6-60.2)	35.6	(31.5-40.0)	29.3	(25.1-33.8)
**30-44 Years**	38.8	(36.7-41.0)	21.1	(19.5-22.9)	16.3	(14.8-17.9)
Low education	34.2	(27.7-41.5)	12.7	(8.9-17.7)	11.1	(7.5-16.1)
Medium education	36.7	(34.0-39.5)	20.2	(18.1-22.5)	15.0	(13.1-17.1)
High education	46.4	(42.9-49.8)	28.1	(25.0-31.3)	22.3	(19.5-25.4)
**45-64 Years**	47.8	(46.0-49.6)	29.4	(27.9-30.9)	22.7	(21.3-24.2)
Low education	44.3	(39.7-49.1)	26.1	(22.5-30.1)	20.0	(16.7-23.7)
Medium education	46.5	(44.3-48.8)	29.3	(27.4-31.2)	22.4	(20.6-24.2)
High education	55.0	(51.5-58.3)	32.7	(30.2-35.4)	26.2	(23.8-28.7)
**≥ 65 Years**	36.5	(34.0-39.1)	26.4	(24.4-28.4)	17.4	(15.6-19.3)
Low education	29.0	(25.6-32.6)	20.7	(17.6-24.1)	12.1	(9.6-15.2)
Medium education	39.4	(35.7-43.2)	28.5	(25.7-31.5)	19.2	(16.6-22.1)
High education	51.1	(45.7-56.5)	38.8	(33.7-44.2)	29.0	(24.4-34.0)
**Total (women and men)**	**45.3**	**(44.2-46.4)**	**29.4**	**(28.6-30.2)**	**22.6**	**(21.8-23.4)**

CI=confidence interval

**Table 2:** Health-enhancing physical activity during leisure time among men according to age and educational status (n=10,448) Source: GEDA 2014/2015-EHIS

Men	Aerobic activity at least 2.5 hours per week		Muscle-strengthening activity at least 2 times a week		Aerobic and muscle-strengthening activity recommendations compliance	
	%	(95% CI)	%	(95% CI)	%	(95% CI)
**Men total**	**48.0**	**(46.6-49.4)**	**31.2**	**(30.2-32.3)**	**24.7**	**(23.6-25.8)**
**18-29 Years**	56.7	(53.6-59.8)	43.9	(41.1-46.8)	35.8	(33.1-38.7)
Low education	52.5	(45.4-59.4)	39.7	(33.5-46.2)	31.4	(25.7-37.8)
Medium education	56.3	(52.4-60.1)	44.9	(41.3-48.5)	36.1	(32.7-39.7)
High education	66.5	(59.7-72.7)	49.1	(42.2-56.0)	43.8	(37.0-50.9)
**30-44 Years**	44.8	(42.1-47.5)	28.5	(26.2-30.8)	22.6	(20.6-24.7)
Low education	36.9	(29.3-45.2)	25.2	(19.0-32.7)	19.9	(14.0-27.3)
Medium education	42.4	(38.7-46.2)	28.2	(25.0-31.6)	22.2	(19.3-25.5)
High education	52.7	(48.9-56.4)	30.6	(27.5-34.0)	24.3	(21.4-27.5)
**45-64 Years**	45.6	(43.7-47.6)	26.3	(24.7-27.9)	21.1	(19.7-22.7)
Low education	35.7	(30.7-40.9)	23.5	(19.2-28.5)	17.9	(13.9-22.8)
Medium education	43.4	(40.8-46.1)	25.3	(23.2-27.6)	20.3	(18.2-22.5)
High education	53.1	(50.3-56.0)	29.1	(26.6-31.8)	23.7	(21.3-26.3)
**≥ 65 Years**	48.3	(45.9-50.7)	32.2	(30.2-34.4)	23.6	(21.6-25.7)
Low education	36.3	(30.9-42.1)	27.3	(22.8-32.4)	18.6	(14.5-23.5)
Medium education	47.2	(43.8-50.7)	30.9	(27.9-34.0)	23.0	(20.1-26.2)
High education	55.2	(51.6-58.7)	36.8	(33.4-40.3)	26.7	(23.7-29.9)
**Total (women and men)**	**45.3**	**(44.2-46.4)**	**29.4**	**(28.6-30.2)**	**22.6**	**(21.8-23.4)**

CI=confidence interval
